# Gating Mechanism for Biased Agonism at Angiotensin II Type 1 Receptors

**DOI:** 10.3390/molecules30112399

**Published:** 2025-05-30

**Authors:** Graham J. Moore, Harry Ridgway, Laura Kate Gadanec, Vasso Apostolopoulos, Anthony Zulli, John M. Matsoukas

**Affiliations:** 1 Pepmetics Inc., 772 Murphy Place, Victoria, BC V6Y 3H4, Canada; 2 THERAmolecular, LLC, Rodeo, NM 88056, USA; ridgway@vtc.net; 3 Institute for Sustainable Industries and Liveable Cities, Victoria University, Melbourne, VIC 8001, Australia; 4 Institute for Health and Sport, Immunology and Translational Research Group, Victoria University, Werribee, VIC 3030, Australia; anthony.zulli@nd.edu.au (A.Z.); imats1953@gmail.com (J.M.M.); 5 School of Health and Biomedical Sciences, RMIT University, Melbourne, VIC 3000, Australia; vasso.apostolopoulos@rmit.edu.au; 6 School of Health Sciences, The University of Notre Dame Australia, Sydney, NSW 2008, Australia; 7 NewDrug PC, Patras Science Park, 26504 Patras, Greece; 8 Department of Chemistry, University of Patras, 26504 Patras, Greece; 9 Department of Physiology and Pharmacology, Cumming School of Medicine, University of Calgary, Calgary, AB T2N 1N4, Canada

**Keywords:** angiotensin II, angiotensin II type 1 receptor, charge relay system, steered molecular dynamics

## Abstract

For the interaction of angiotensin II (AngII) with AngII type 1 receptors (AT_1_R), two potential proton hopping pathways have been identified, each associated with distinct physiological outcomes. The octapeptide AngII (Asp^1^-Arg^2^-Val^3^-Tyr^4^-Ile^5^-His^6^-Pro^7^-Phe^8^) appears to form a charge relay system (CRS) in solution in which the C-terminal carboxylate abstracts a proton from the His^6^ imidazole group, which, in turn, abstracts a proton from the Tyr^4^ hydroxyl (OH) group, creating a tyrosinate anion. When AngII binds to the AT_1_R, the CRS can be reconstituted with D281 of the receptor taking up the role of the Phe^8^ carboxylate in the tripartite interaction, whilst the Phe^8^ carboxylate forms a salt bridge with K199 of the receptor. As a consequence, the Tyr^4^ OH of AngII is positioned with accessibility to either the Phe^8^ carboxylate (bound to K199) or the His^6^ imidazole (activated by D281), thereby creating a potential gating mechanism for AT_1_R receptor signaling. This study summarizes evidence based on structure activity data for various analogs wherein Tyr^4^ OH interaction with His^6^ imidazole (CRS formation) leads to G protein sequestration and vasoconstriction, whereas Tyr^4^ OH interaction with Phe^8^ carboxylate (bound to K199) engenders arrestin-mediated vasodilation and receptor desensitization. These findings, combined with quantum mechanical (semiempirical) calculations of CRS proton transfer presented herein, provide insights for the therapeutic targeting of angiotensin receptor blockers (sartans) and the development of second-generation drugs (bisartans).

## 1. Introduction

The renin–angiotensin system (RAS) is a complex hormonal system that is pivotal for blood pressure homeostasis and fluid balance because it controls hemodynamic flow and volume, electrolyte absorption and excretion, and vascular resistance and tone [1,2]. The effects of the RAS are facilitated by highly sophisticated biomolecular cascades involving enzymatic cleavage and the alteration of pro-hormones and intermediary proteins and other bioactive compounds that exert the majority of their actions through the activation of G protein-coupled receptors [3,4]. The intricacy of the RAS is further highlighted by the existence of two distinct and opposing axes within cells, glands, and organs that provide autocrine, paracrine, and systemic mechanisms to maintain cardiovascular and renal physiology [5,6]. The classical axis is considered the main effector pathway and is primarily mediated by angiotensin-converting enzyme (ACE), angiotensin II (AngII), and AngII type 1 receptor (AT_1_R) [7,8]. In contrast, the alternative arm is responsible for counter-regulating the effects of the classical RAS, largely due to the actions of angiotensin-converting enzyme-2, angiotensin 1-7, and Mas1 proto-oncogene receptor [9,10]. 

**Figure 1:**
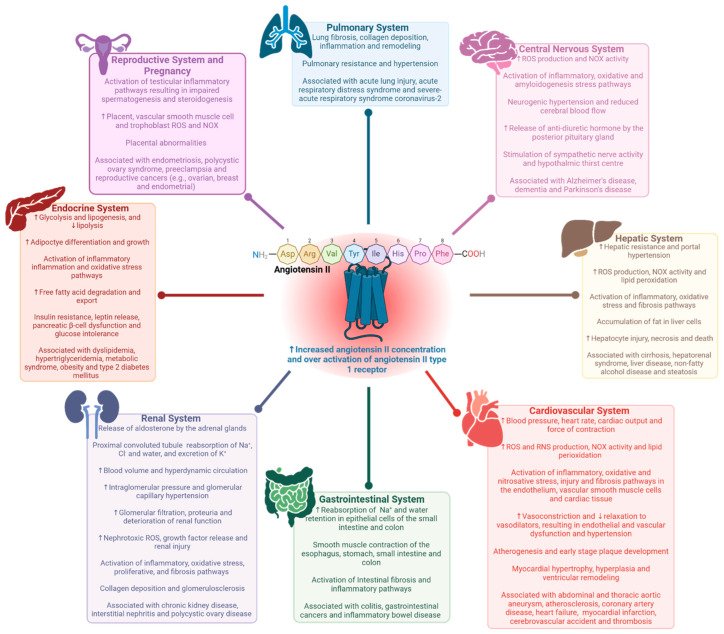
Pathological processes and diseases associated with the dysregulation of AngII and overactivation of AT_1_R. Increased levels of AngII and subsequent overstimulation of AT_1_R have clinically been correlated with deleterious processes and diseases involving the cardiovascular [7,10,11,14], central nervous [15,16], endocrine [17,18,19,20], gastrointestinal [1,21], hepatic [22,23], pulmonary [24], renal [10,25], and reproductive [26,27,28] systems. Abbreviations: AngII, angiotensin II; Arg, arginine; Asp, aspartic acid; AT_1_R, angiotensin II type 1 receptor; Cl^−^, chloride; His, histidine; Ile, isoleucine; K^+^, potassium; Na^+^, sodium; NOX, nicotinamide adenine dinucleotide phosphate oxidase; Phe, phenylalanine; Pro, proline; RNS, reactive nitrogen species; ROS, reactive oxygen species; Tyr, tyrosine; Val, valine. Key: C, carbon (black); H, hydrogen (grey); N, nitrogen (blue); O, oxygen (red); ↑, increase; ↓, decrease. Figure created using Biorender.com (accessed on 1 February 2025).

**Figure 2:**
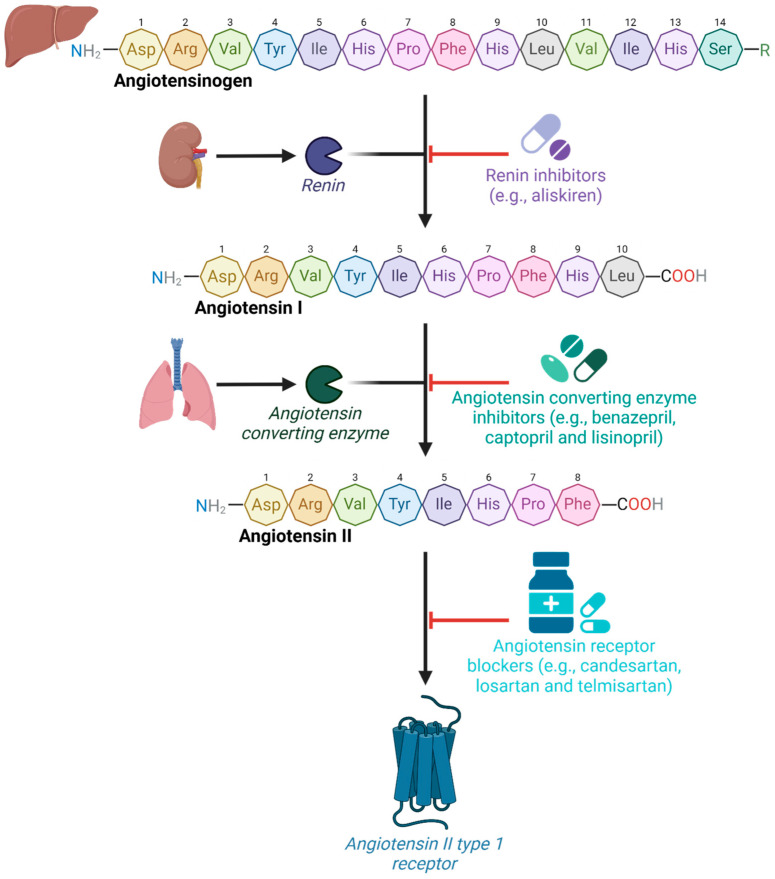
Peptides and inhibitors of the classical axis of the RAS. Angiotensinogen is catalyzed by renin, an aspartic protease produced by renal juxtaglomerular cells [29], to the relatively biologically inactive decapeptide, AngI [29]. AngI is then converted to AngII by ACE, which primarily exerts its effects by associating with AT_1_R [7,8]. Dysregulation of the classical axis has been well established as a major contributor underlying cardiovascular and renal diseases [12,13,30]. Medications that target and inhibit components at each step in the classical cascade, including renin inhibitors, ACE inhibitors, and ARBs, have been created to restore homeostatic balance [12,13,30]. Abbreviations: ACE, angiotensin-converting enzyme; ARB, angiotensin receptor blockers; AngI, angiotensin I; AngII; angiotensin II; Arg, arginine; Asp, aspartic acid; AT_1_R, angiotensin II type 1 receptor; His, histidine; Ile, isoleucine; Leu, leucine; Phe, phenylalanine; Pro, proline; RAS, renin angiotensin system; Ser, serine; Tyr, tyrosine; Val, valine. Key: C, carbon (black); H, hydrogen (grey); N, nitrogen (blue); O, oxygen (red); R, sidechain (green). Figure created using Biorender.com (accessed on 1 February 2025).

AngII (Asp^1^-Arg^2^-Val^3^-Tyr^4^-Ile^5^-His^6^-Pro^7^-Phe^8^), generated by angiotensin-converting enzyme acting on angiotensin I (AngI), is an octapeptide hormone considered to be the principal pleiotropic effector of the RAS [11]. Under normal circumstances, AngII maintains physiological control by predominantly interacting with AT_1_R to stimulate anti-diuretic hormone and aldosterone secretion, sodium reabsorption, water retention, sympathetic nervous system activation, and vascular smooth muscle contraction [11]. However, the unregulated synthesis and release of AngII resulting in overactivation of AT_1_R has clinically been linked to pathological processes, including endothelial and vascular dysfunction, inflammation, oxidative stress, fibrosis, hypertrophy, and the development of diseases involving multiple organ systems (Figure 1). Medications, such as renin inhibitors [12], ACE inhibitors, and angiotensin receptor blockers (ARBs) [13], are commonly prescribed medications to reduce the detrimental cardiovascular and renal effects of classical RAS dysregulation (Figure 2).

Structure–activity relationships have been developed for AngII, which demonstrate that the replacement of the C-terminal Phe^8^ residue with aliphatic amino acids, such as alanine (i.e., saralasin) or isoleucine (i.e., sarilesin), results in inverse agonist activity at AT_1_R (which is accompanied by the desensitization of the receptor) [31,32,33,34]. Thus, this implies that the Phe^8^ aromatic ring has a critical role in the contractile mechanism for AngII [32,33,34]. Agonist (contractile) effects of AngII are mediated by G protein, whereas inverse agonist effects (relaxation, which is accompanied by the desensitization of receptors) are mediated by beta-arrestin (β-arrestin) [35,36,37,38,39]. Inverse agonists are known to evoke a desensitization effect that is similar to tachyphylaxis [40], which may be due to receptor internalization [38,41].

AT_1_R, like many other G protein-coupled receptors, exists in dimer form within the cell membrane, allowing agonists to invoke homotropic cooperativity (accompanied by heterotropic cooperativity due to interaction with G protein) [42,43,44,45], such that the agonist can induce an increase in its own affinity and thereby amplify the response over a narrow concentration range [45]. Conversely, very high concentrations of an agonist or the presence of an inverse agonist appear to invoke negative cooperativity, and the receptor switches from coupling to G protein to coupling to arrestin [45]. It is not clear if the mechanism for switching is due to the binding of AngII to a tertiary agonist binding site on the receptor or if it results from an unidentified signaling molecule becoming operative when an upper limit response threshold is achieved. The receptor also becomes refractory to the agonist for extended time periods (i.e., desensitization and/or tachyphylaxis), possibly due to receptor internalization [38,41,45].

Herein, this study utilizes the structure–activity molecular mechanism of receptor AT_1_R interactions and ex vivo isometric tension analysis studies to explain agonist and inverse agonist behaviors.

## 2. Results and Discussion

### 2.1. Charge Transfer Interactions in AngII Before and After Receptor Binding

**Figure 3:**
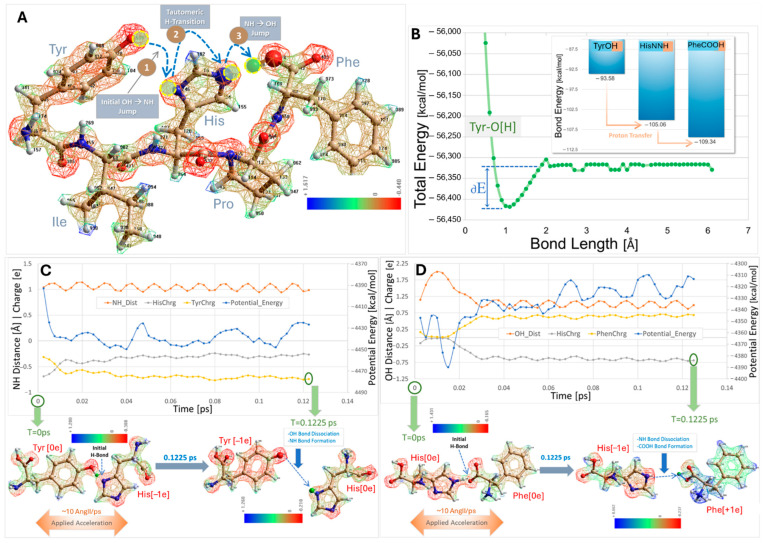
(**A**) AngII structure optimized in vacuo using a UHF/RM1 semiempirical QM method (0.1 kcal/mol convergence) showing CRS proton transfer (arrows). Electrostatic charge distribution of AngII is depicted as a wire-mesh: red = negative; green = neutral; blue = positive. (**B**) Hydrogen-dissociation energy calculations for the three neutral CRS species in AngII: Tyr^8^OH^0e^, His^6^NNH^0e^ and Phe^8^COOH^0e^ (see Methods). Results indicate favorable proton transfer from Tyr hydroxyl to His imidazole to Phe carboxylate (see text for details). (**C**,**D**) Non-equilibrium steered MD (SMD) calculations (UHF/RM1; see Methods) for CRS hydrogen-bonded reaction pairs: TyrO[H]^0e^---NNHis^−1e^ (**C**), and HisNN[H]^0e^---OOCPhe^−1e^ (**D**). Results were in overall agreement with the H-dissociation energies calculated in B and the proposed CRS mechanism (see text for details). Abbreviations: AngII, angiotensin II; His, histidine; HisNNH^0e^ and His6NN^−1e^ (or NNHis^−1e^), protonated or non-protonated His imidazole nitrogen; Ile, isoleucine; Phe, phenylalanine; Pro, proline; SMD, steered molecular dynamics; Tyr, tyrosine; TyrOH^0e^ or TyrO^−1e^, protonated or non-protonated phenol OH group of Tyr; Å, Angstrom.

Semiemprical energy calculations conducted (in vacuo) on paired interactions of the three residues comprising the charge relay system (CRS) (Tyr, His, Phe) have shown that the overall energy gradient favors proton transfer from Tyr^4^OH to His^6^ imidazole to the Phe^8^ carboxylate (see Figure 3), but that there is a possible slight uphill gradient for proton transfer from His^6^NNH^0e^ imidazole to Phe^8^COO^–1e^ carboxylate (Figure 3D). It is possible that other groups in the AngII peptide have a role in assisting the transfer of the Tyr^4^OH proton to His^4^ imidazole resulting in the tyrosinate anion species detected by spectroscopy. Indeed, a role for the Arg^2^ guanidinium sidechain in chaperoning this process has been speculated on previously [46]. Quantum mechanical (semiempirical RM1) calculations of the hydrogen dissociation energies for TyrOH, HisNH imidazole, and PheCOOH, carried out by computing the optimized residue energy as a function of the OH (Tyr and Phe) or NH (His) bond lengths were carried out. In each case, the bond energies obtained from the energy/bond length plots were the differentials between the lowest energy observed compared to the energy at “infinite” hydrogen separation (delta-E in Figure 3B). The result is that the order of the hydrogen dissociation energies is Phe > His > Tyr, such that Tyr will give up its proton to His, and His to Phe, in accordance with the proposed CRS mechanism.

The details of the energetic considerations of the AngII CRS in Figure 3 are as follows: (A) A local-energy-minimized (semiempirical RM1/UHF-optimized, 0.1 kcal/mol convergence) conformation of human AngII with labeled residues. The electrostatic potential distribution (calculated in Hyperchem software, Version 8.01) is rendered in a wire mesh with partial atomic charges ranging from blue (positive) to green (neutral or intermediate) to red (negative). The diagram indicates one possible route for charge (proton) transfer from the TyrOH^0e^ to HisNN^−1e^ (Step 1) to PheCOO^−1e^. A key aspect of this CRS model is a tautomeric transition of the donated proton from TyrOH^0e^ to the deprotonated (activated) acceptor HisNN^−1e^ (Step 2). (B) Bars indicate relative OH or NH bond energies for TyrO[H], HisNN[H], and PheCOO[H]. Bond energies were calculated (RM1,/UHF) from bond distance versus energy plots for each neutral optimized species in vacuo. The inset in B shows the distance/energy plot for the Tyr hydroxyl O[H] bond. Plots for the N[H] bond of neutral His and for the COO[H] bond of Phe were similar. In each case, the bond energy was obtained from the difference between the minimum energy observed (at the ideal bond length of about 1.1 Å) and the energy at “infinite” separation (i.e., after the OH or NH covalent bond was completely broken, resulting in no further change in the energy profile). This generally occurred at around 2–3 Å of separation. Bond energies varied from about 94 kcal/mol for the OH bond of TyrOH to 109 kcal/mol for the OH bond of PheCOOH and were similar to experimentally reported energies (https://www2.chemistry.msu.edu/faculty/reusch/OrgPage/bndenrgy.htm) (accessed on 1 April 2025). The computed bond energies were consonant with a CRS mechanism involving a thermodynamically favorable proton transfer from TyrOH^0e^ to HisNN^−1e^ to PheCOO^−1e^ (C and D). An independent calculation method in which paired hydrogen-bonded residues (e.g., TyrOH---NNHis) were “pulled” apart using non-equilibrium steered molecular dynamics simulations (SMDs) to determine which residue partner received the shared proton also supported the Tyr → His → Phe pathway mechanism. (C) SMD trajectory analysis (in vacuo; RM1/UHF calculation) indicating proton migration from TyrOH^0e^ to HisNN^−1e^. The paired residues (at T = 0 ps) were separated by about 2.5 Å following an initial RM1/UHF geometry optimization and they interacted via a single hydrogen bond (TyrO[H]---NNHis). A directional (vectored) acceleration bias of 10 Å/ps was applied to the His residue, thereby pulling it away from Tyr and causing the TyrOH proton to be abstracted, creating a tyrosinate anion (TyrO^−1e^) and the neutral HisNNH^0e^ species; hence, TyrOH^0e^ + HisNN^−1e^ → TyrO^−1e^ + HisNNH^0e^. The trajectory analysis associated with the deprotonation reaction in C shows that the subsequent NH covalent bond distance of HisNNH^0e^ exhibited a sine wave fluctuation (a likely historesis effect) due to non-dissipated kinetic (thermal) input into the system. Additionally, the net residue charges for Tyr and His (computed from the summation of their respective partial atomic charges) diverged as expected while the molecular separation progressed, with Tyr becoming more negative (yellow line) and His becoming more positive (gray line). Charge divergence and residue separation were accompanied by a net decline in total system potential energy (blue line). (D) SMD reaction simulation similar to that described in (C), except using the hydrogen-bonded pair, namely HisNN[H]---OOCPhe. In this case, the HisNNH^0e^ proton was abstracted and underwent covalent bond formation with PheCOO^−1e^ to form neutral PheCOOH^0e^, with an inital abrupt energy decrease during the first 0.02 ps. While a slight increase in overall system potential was observed (from conformational instability due to kinetic perturbation), the newly formed OH covalent bond distance fluctuations of PheCOOH^0e^, and net residue charges exhibited the same overall trends as were observed for the TyrOH^0e^/HisNN^−1e^ reaction pair described above in (C).

**Figure 4:**
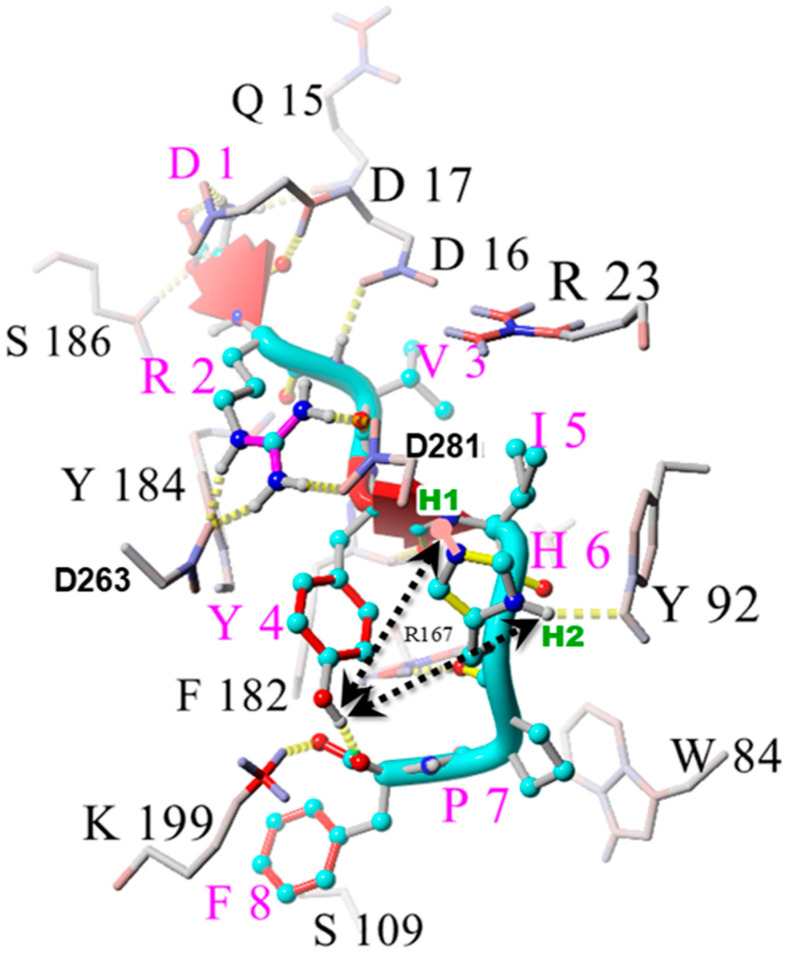
X-ray crystallographic conformation of the AngII oligopeptide extracted from the AT_1_R G protein-coupled receptor (PDB 6OS0; [37]). AngII residue labels are colored magenta while AT_1_R residue labels are black. Intermolecular hydrogen bonds are indicated as thick dotted yellow lines. Atom colors: cyan = C; blue = N; gray = H; for K199: red = N, blue = H. The conformation depicts a number of putative interactions between AT_1_R and AngII that regulate GPCR signal transduction through the CRS pathway. For example, both Asp^1281^ (D281) and Asp^1263^ (D263) of AT_1_R form a stabilizing H-bonded cage-like network with R^2^ of AngII. The H^1^ and H^2^ hydrogen positions of H^6^ (green bold labels) represent equilibrium tautomeric forms of neutral H^6^, and experimental evidence indicates that H^6^ participates in the CRS by accepting a proton donated by the Y^4^ terminal -OH group forming tyrosinate anion. Depending on a number of possible conformational changes in AngII, Y^4^ may relinquish its proton to either of the H^6^ tautomeric forms, as indicated by the black dotted lines. It is also possible that Y^4^ can donate its proton directly to the –COO group of F^8^ by means of a direct hydrogen bond, as suggested in the X-ray conformation shown in the figure. The H^6^ imidazole can also be activated by the abstraction of the imidazole proton by D281, creating a possibility for the Tyr^4^-OH [shown H-bonded to Phe^8^ carboxylate] to move rightwards ~6 Å to the non-protonated imidazole nitrogen of H^6^ and form the CRS. Note that the crystal structures of AngII and saralasin are known to differ only with respect to Arg^2^ interactions [37], which are with D263 for AngII and with D263 and D281 for saralasin. Thus, the conformation shown here highlights the principal interactions of AngII which would likely prevail [in the absence of G protein binding] in inverse agonist binding mode (i.e., tachyphylaxis), where R^2^ interacts with D281 in addition to D263. Abbreviations: AngII, angiotensin II; AT_1_R, angiotensin II type 1 receptor; CRS, charge relay system; D, aspartic acid; F, phenylalanine; H, histidine; H-bond, hydrogen bond; K, lysine; Q, glutamine; R, arginine; S, serine; W, tryptophan; Y, tyrosine; Å, Angstrom.

**Figure 5:**
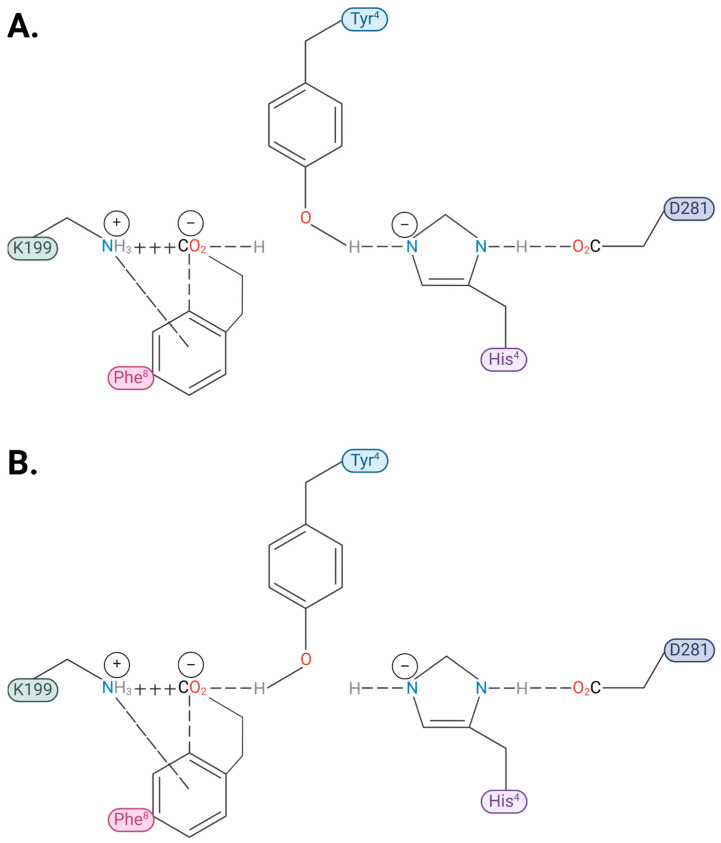
Gating mechanism for biased agonism at AT_1_R. Tyr^4^OH acts as a molecular switch which can swing between two poles, depending on the relative pKa of the two poles [as determined by the interactions of Arg^2^ (see text)]. (**A**) Agonist activity is engendered by the formation of a CRS wherein His^6^ imidazole is activated by D281. (**B**) Inverse agonist activity results when Tyr^4^ OH forms an H-bond with the C-terminal carboxylate [in a salt bridge with K199]. The single-letter code for amino acids denotes receptor-based residues. Note that for figurative purposes aromatic rings are not shown in their true orientations (see text for details). Abbreviations: Arg, arginine; C, carbon (black); H, hydrogen (grey); H-bond, hydrogen bond; His, histidine; N, nitrogen (blue); O, oxygen (red); Phe, phenylalanine; Tyr, tyrosine. Figure created using Biorender.com (accessed on 1 February 2025).

Several lines of evidence, accumulated from two-dimensional rotating frame nuclear Overhauser effect spectroscopy, tyrosinate fluorescence lifetime spectroscopy [47], and chemical reactivity and catalytic property studies [48], have reported that the solution conformation for AngII contains a CRS. The CRS is similar to that found at the active site of serine (Ser) proteases, by which Ser is replaced by tyrosine (Tyr) to form Tyr hydroxyl (OH)- -histidine (His)- -carboxylate (Figure 3). This CRS interaction produces a tyrosinate anion, which appears to be important for receptor activation [47,49]. The crystal structure for the AT_1_R bound to AngII shows that the CRS found in solution can be reconstructed at the receptor, with D281 supplanting the role of the C-terminal carboxylate of AngII by interacting with His^6^ imidazole, enabling the recreation of the tripartite interaction [37]. In its receptor-bound form, the Tyr^4^OH of AngII is positioned to swing between binding to either the C-terminal carboxylate of AngII (present in a salt bridge with K199 of the receptor) or the His^4^ imidazole (activated by D281 of the receptor), providing a possible molecular switch or gating mechanism for receptor signaling events [37,50] (Figure 4 and Figure 5). Interestingly, the CRS in AngII shown in Figure 3 exists sparingly in aqueous solution but becomes the dominant conformer in the dehydrated low dielectric receptor environment [47], and is apparently preconditioned or “setup” for the prescribed interaction with the functional groups at the active site of the AT_1_R (Figure 4 and Figure 5). It is possible that other known “tyrosinate” hormones (e.g., vasopressin, oxytocin, gonadotrophin-releasing hormone, and enkephalin) which also require the formation of tyrosinate for activating their respective receptors may take up prescribed conformations in a similar manner to AngII, which is predisposed for receptor binding [51].

The Phe^8^ aromatic ring in AngII plays a critical role in the expression of the agonist activity of AngII, since its substitution by alanine or isoleucine results in peptides (saralasin, sarilesin) which are inverse agonists. The crystal structures of AT_1_R bound to AngII, and also bound to saralasin [37], have provided insights into receptor mechanisms. AngII binds to AT_1_R in an extended conformation in which the main contacts providing for affinity are with aspartyl sidechains on the receptor, namely D17 with N-terminal amino, D263 with Arg^2^ guanidinium, and D281 with His^6^ imidazole (Figure 4). In addition, K199 forms a salt bridge with the C-terminal carboxylate of AngII, while Tyr^4^ OH is positioned for interactions with either of the anionic sites provided by (1) the C-terminal carboxylate (bound to K199) forming an H-bond, or (2) His^6^ imidazole (activated by D281), thereby forming a CRS. In Figure 4, the phenolic proton is shown bound (H-bond) to the C-terminal carboxylate of AngII, but it can potentially move ~5 Angstrom (Å) to the right (viewing this figure frontally) and interact with the (D281-activated) His^6^ imidazole unprotonated nitrogen (thereby forming the equivalent CRS interaction seen for AngII when free in solution). Figure 5 elaborates upon this theme in detail and explores how these two scenarios could lead to agonism and inverse agonism, respectively.

The absence of the Phe^8^ aromatic ring in saralasin leads to upstream conformational changes in the peptide at the receptor wherein the Arg^2^ guanidinium group, which does not interact significantly with D281 in AngII [37], binds to D281 in addition to D263 in saralasin [11], thereby weakening the interaction of D281 with the His^6^ imidazole, and decreasing the likelihood of CRS formation. Thus, the interaction of the Phe^8^ aromatic ring with the salt bridge appears to have a direct influence on the positioning and interactions of the Arg^2^ guanidinium group, which can bond exclusively to D263 in AngII, allowing D281 to interact exclusively with His^6^ imidazole. Note that in Figure 4 the binding mode for AngII, which includes interactions of Arg^2^ with both D263 and D281, is thought to represent the binding of AngII in the inverse agonist mode (see legend).

Ring currents present in aromatic rings create a quadrupole moment in which there is a negative charge above and below the plane of the ring and a positive charge in the plane of the ring [52]. Accordingly, the Phe^8^ aromatic ring forms a dual interaction with the salt bridge formed between the C-terminal carboxylate and K199, in which the out-of-plane negative charge interacts with the amino group of K199 whilst the in-plane positive charge interacts with the carboxylate, thereby placing the ring in a vertical position (Figure 4). Note that, in Figure 5, the Phe^8^ ring, which is pictured horizontally to show quadrupole interactions, would in reality exist in a vertical position as in Figure 4. Notably the Phe^8^ ring cannot simply rotate/flip on its axis through 90 degrees and adopt a static horizontal placement, because of its quadrupole moment, but the ring is able to roll (like the rim of a wheel around an axle, or axis of the salt bridge) to a position above or below the axis of the salt bridge (assuming no steric constraints). Quadrupole interactions are weak, and the energy change for rolling from vertical to horizontal (<1 kcal/mole) is unlikely to provide the signal-to-noise ratio required for an important switch in mechanism by the receptor—especially given the available CRS alternative where a change in charge status (resulting from protonation of D281) is offered by the CRS mechanism. However, binding of the Phe^8^ ring to the K199 salt bridge (estimated energy gain ~5 kcal/mole) appears to be sufficient to invoke a conformational change in the peptide–receptor complex, in turn allowing Arg^2^ guanidinium to direct its interaction exclusively to one salt bridge (D263), so that it does not interfere with D281’s interaction with His^6^ imidazole and the potential for CRS formation.

In essence, the Tyr^4^ OH of AngII when bound to AT_1_R is potentially able to interact with either the C-terminal carboxylate, which is virtually neutralized (partially “protonated”) by K199, or with the His^6^ imidazole, which acts as an electrical conduit for the carboxylate of D281 (Figure 4 and Figure 5). The stronger interaction (lower apparent pKa) could be with the pole represented by the His^6^ imidazole (CRS switch) because of the availability of the formal proton transfer, and, in the absence of mitigating circumstances which impair the acidity of the His^6^ pole (such as the additional bonding of Arg^2^ to D263 seen for saralasin), agonist activity will be favored. It is worth noting that for the CRS interaction, the two rings (Tyr^4^ and His^6^) would prefer to take up a particular geometry (see legend to Figure 5), in which the transitioning negative charge on the imidazole ring (produced by interaction with D281) will form a perpendicular plate ring–ring interaction in which the in-plane positive charge on the Tyr ring interacts with the flat cationic surface of the His imidazole ring.

As outlined above, the interaction of the Phe^8^ aromatic ring quadrupole with the salt bridge appears to influence the positioning and interactions of the Arg^2^ sidechain and may explain why electrostatic disruptions to the geometry of its interaction with D263 (AngII) versus D263 plus D281 (saralasin) can lead to changes in agonist versus inverse agonist behavior. For example, replacement of the Phe^8^ ring with a polyfluorinated ring (F5-Phe), which inverts the ring quadrupole [53], will cause a change in the geometry [the ring will relocate so that the positive charges above and below the plane of the ring can interact with the carboxylate, while the negative charge in the plane of the F5-ring interacts with the K199 amino group]. This changed ring geometry [from vertical to horizontal] results in mixed agonist/inverse agonist behavior [53], likely because there has been a corresponding effect on Arg^2^ interactions with D281 [relative to AngII binding] which has resulted in weakening of the CRS, thus imbuing partial inverse agonist behavior. The substitution of a cyclohexyl ring for the Phe ring produces mixed effects including mild agonist properties [54], which contrasts with the purely inverse agonist effects of isosteric sarilesin, probably because aliphatic rings can also induce weak ring currents (e.g., graphene), thereby retaining some of the characteristics of an aromatic ring. Similar considerations can be applied to an analog in which the Phe^8^ ring is forced to adopt a vertical orientation at the K199 salt bridge by virtue of a covalent methylene bond between the Phe ring and the alpha carbon of the amino acid, which results in inverse agonism [36]. In our model (Figure 5), the vertical positioning of the ring might be expected [taken at simplistic face value] to lead to CRS formation and agonist activity, but other accompanying electrostatic and geometric changes for this analogue may be sufficient to alter the balance, resulting in a switch towards inverse agonism. Every peptide chemist will attest that profound changes are brought about when the alpha C-proton of an amino acid is substituted, including changes to the reactivity of both carboxylate and amido functionalities, in addition to steric hindrance effects.

Peptides, like saralasin and sarilesin, which are potent inverse agonists, likely owe this property largely to the influence of Arg^2^ guanidinium interacting with D281 and preventing CRS formation, which would otherwise provide for agonist activity. Accordingly, it might be predicted that if the Arg^2^ residue was replaced with Ala^2^ in sarilesin, then the disruptive interaction with D281 could no longer take place, and the resulting peptide (Sar^1^ Ala^2^ Ile^8^-AngII) should demonstrate measurable agonist activity, and indeed this is the case [36]. On the other hand, removal of the guanidinium sidechain of AngII [as in Sar^1^Ala^2^ -AngII] results in a marked decrease in agonist activity. Apparently, while the Phe^8^ ring appears to influence the positioning of Arg^2^ so that it does not interact effectively with D281, the guanidinium group may, in turn, influence the [salt bridge] interactions of the Phe^8^ ring and, therefore, the agonist activity. Since these two groups do not appear close enough for direct interaction (Figure 4), reciprocal influences are presumably transmitted through the backbone structure, possibly in concert with pole-to-pole motion of the Tyr^4^ sidechain (Figure 5).

CRS interaction at the receptor results in the formation of tyrosinate anion [36], which appears to be required to drive the cooperative receptor dimer interaction which increases the agonist affinity and amplifies the response mechanism. More importantly, tyrosinate formation implies that the carboxylate of D281 has been effectively neutralized by CRS formation (the TyrOH proton has been transferred to the D281 sidechain), and that this change in the charge on this receptor-based aspartyl residue could provoke a conformational change in the receptor sufficient to induce cooperativity and activate G protein signaling. In contrast, inverse agonism appears to be associated with a lower energy hydrogen bond interaction between Tyr^4^ OH and the Phe^8^ carboxylate of the salt bridge [which is absent formal proton transfer]. This H-bond interaction, rather than the CRS interaction of Tyr^4^OH with D281-activated His^6^, is observed in the crystal structure for AngII bound to AT_1_R, notably even when there is clearly no Arg^2^ interaction with D281 [51], despite the CRS being energetically favored. This apparent anomaly may be due to the absence of G protein, which did not co-crystallize with AT_1_R, possibly preventing cooperative formation of the CRS option. Note that the structure shown in Figure 4, wherein Arg^2^ interacts with both D263 and D281, may represent the binding of AngII in inverse agonist mode (tachyphylaxis, see legend to Figure 4 for details).

In this model there is a potential role for Arg^2^ guanidinium as a gatekeeper because its interaction, or not, with D281 would determine inverse agonist versus agonist activity, respectively. However, the formation of a salt bridge between Arg^2^ guanidinium and D281 also appears to depend on the absence of the Phe^8^ aromatic ring, because it forms in saralasin but not in AngII [37]. The interaction of Tyr^4^OH within the CRS [at the receptor in the presence of facilitatory groups] could be stronger (proton transfer) than with the Phe8 carboxylate partially neutralized in the salt bridge (H-bond) and may, therefore, be energetically favored [so long as Arg^2^ does not interact with D281]. Apparently conformational changes in the immediate receptor vicinity invoked by the Phe^8^ aromatic ring interaction within the salt bridge are able to change the positioning of the Arg^2^ guanidinium group relative to D281. When AngII binds in agonist mode there is no interaction of Arg^2^ guanidinium with D281 [37] (the interaction is exclusively with D263, allowing CRS formation). Dual interactions of Arg^2^ with D281 and D263 are prevalent only for inverse agonist peptide analogs, like saralasin (thereby preventing CRS formation), and may also occur for AngII in inverse agonist mode (Figure 4). Tachyphylaxis to AngII may result from a feedback mechanism which causes Arg^2^ to interact with D281, as in Figure 4, thereby inhibiting CRS formation and causing reversion to H-bonding of the Tyr^4^ OH with the Phe^8^ carboxylate of the salt bridge, producing inverse agonism with consequential desensitization.

### 2.2. Analysis by Amino Acid Position in AngII

**Position 1:** It is well established that the substitution of sarcosine (Sar) at position 1 increases the potency of all AngII analogs, including agonists (Sar^1^-AngII is a superagonist), competitive antagonists, like Sar^1^Tyr(OMe)^4^-AngII (sarmesin), and inverse agonists (i.e., saralasin and sarilesin). This is not due to increased metabolic stability but likely originates from the removal of a distracting interaction of the amino terminal with the Asp sidechain (normally present in AngII), allowing increased interaction of the amino terminus with D17 of the receptor (Figure 5). Also, since Gly^1^ and Ala^1^ substitutions result in weaker activities than Sar^1^, the fact that Sar is a secondary amino acid (and, therefore, a stronger base) is probably relevant.

**Position 2:** Arg guanidinium forms a salt bridge with D263 of the receptor (Figure 4), thereby contributing to receptor binding affinity. In saralasin, Arg^2^ guanidinium also interacts with D281, weakening CRS formation and thereby engendering inverse agonist properties [but notably Ala^2^ substitution restores some agonist activity to sarilesin].

**Position 3:** Val is a sterically hindered amino acid which creates a turn in the peptide backbone which is essential for activity.

**Position 4:** Tyr OH can function as a molecular switch (Figure 5), operating by binding to either of two acid groups according to their relative availability/acidity: interaction with His^6^ imidazole (activated by D281) appears to result in agonist activity, whereas interaction with C-terminal carboxylate (bound to K199) produces inverse agonist activity. Methylation of the Tyr hydroxyl of AngII inhibits receptor signaling for both agonist and inverse agonist analogs, such that Sar^1^Tyr(Me)^4^-AngII (sarmesin) is a competitive antagonist, while Sar^1^Tyr(Me)^4^Ile^8^-AngII is essentially inactive.

**Position 5:** Ile is a sterically hindered amino acid which creates a turn in the peptide backbone which is essential for activity.

**Position 6:** His imidazole is essential for bioactivity and engages in an interaction with D281 which acts as one pole of the gating mechanism leading to agonist activity via CRS formation (see Figure 5).

**Position 7:** Pro is a sterically hindered amino acid which creates a gamma turn in the peptide backbone, which is essential for activity [and which enables the C-terminal carboxylate to engage with His^4^ and also with the Phe^8^ ring in solution [46] (Figure 3), setting the stage for specific interactions at the receptor (Figure 4 and Figure 5).

**Position 8:** The Phe aromatic ring is a critical determinant of agonist activity due to its quadrupolar interactions with the salt bridge formed between the C-terminal carboxylate of AngII and K199 of the receptor, resulting in repositioning the Arg^2^ sidechain to interact exclusively with D263 and not with D281, which interacts exclusively with His^6^. Elimination or substitution of the Phe^8^ aromatic ring [as in saralasin and sarilesin, respectively] produces inverse agonism [Arg^2^ interacts with D281 and compromises formation of the CRS], whereas replacement with F5-Phe [inverted quadrupole] or cyclohexyl [weak ring current] produces mixed agonist/inverse agonist effects (partial agonists [45]), due to electrostatic and geometric changes influencing the interaction at the salt bridge and consequential effects on Arg^2^ interactions.

### 2.3. AngII Causes Biphasic Vasoactive Responses in Mouse and Rat Iliac Arteries

**Figure 6:**
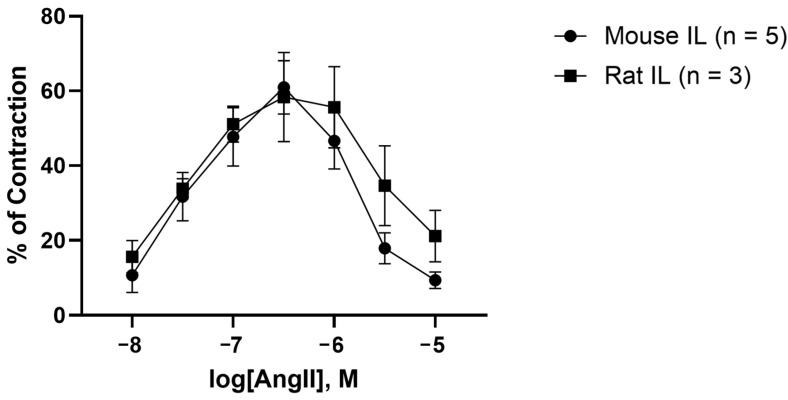
AngII-induced contraction responses in mouse and rat iliac arteries. An AngII dose–response test caused biphasic vasoactive responses (mean ± SEM), which involve contraction responses followed by reduction contraction and relaxation. Abbreviations: AngII, angiotensin II; IL, iliac artery; SEM, standard error of mean.

An AngII dose–response test invoked biphasic vasoactive responses in mouse and rat iliac arteries which resemble a “bell” or parabola shape (Figure 6). Furthermore, we demonstrate that the vasocontraction responses to AngII are not dose-dependent, as the greatest effect (i.e., percentage of contraction) or vertex of the graph does not correlate to the highest concentration of AngII. Alternatively, the greatest effects of AngII in both mice (60.94 ± 7.16%) and rat (58.39 ± 12.00%) iliacs were observed at 10^−6.5^ M (Figure 6). Moreover, there was no significant differences in contraction responses between mouse and rat iliac arteries. These results support previous findings from our and other laboratories that report the ability of AngII to induce a biphasic response to AngII in human [55,56,57], mouse [58,59,60,61,62], rabbit [63,64,65,66], and rat [67,68,69] arteries (Table 1). This two-stage process (contraction followed by reduced contraction and relaxation) implies that AngII switches from agonism to inverse agonism effects at AT_1_R and may be due to recruitment of β-arrestin [36,37,38,39,40], receptor desensitization [32,33,34,35] and internalization [70,71], and/or tachyphylaxis [41,72]. Conversely, AngII has also been shown to cause contraction responses that are dose-dependent [73,74,75,76,77,78,79], resulting in a sigmoidal shaped graph where the greatest AngII-induced contraction corresponds to the highest AngII concentration (Table 1). These discrepancies in AngII-mediated vasoactive responses may depend on artery type, the expression profile of AT_1_R, genetic variations/genotype and/or disease state, requiring a greater concentration of AngII to overcome the threshold to trigger inverse agonistic effects.

### 2.4. Tachyphylaxis, Receptor Desensitization, and Inverse Agonism by ARB Sartans

Tachyphylaxis is a well-established phenomenon in which excessive concentrations of agonist desensitize the receptor to that agonist, presumably offering a protective mechanism against the overstimulation of cells. Certain analogs of AngII, which have no agonist activity (e.g., saralasin and sarilesin), can induce the same effect by desensitizing isolated tissues for extended periods, presumably by a similar mechanism. Thus, AngII has agonist activity at physiological concentrations but appears to act as an inverse agonist at supramaximal concentrations. In isolated tissues, the presence of inverse agonist, or of excess agonist, produces the deactivation of receptors, which is similar to that produced by a chemically irreversible ligand, giving the impression that receptors effectively disappear. Indeed, this may well be the case, because receptor internalization is an established phenomenon, and AngII receptors do not change or disappear in membrane preparations from the corresponding isolated tissue. The time delay for the re-sensitization of receptors (<1 h in isolated tissue assays) is probably too short for synthesizing new receptors but may be consistent with the shuttling of pre-synthesized receptors from an intracellular storage depot to the cell membrane.

It is not clear whether the tachyphylaxis switches from agonism to inverse agonism seen for AngII at high concentrations is mediated by a tertiary binding site, or by changes in downstream signaling factors (e.g., exhausting the supply of G protein cofactors). It is possible that when the maximum response to AngII is achieved (receptor saturation by the agonist), there are changes in the underlying conformation of the receptor which enables AngII to bind, like saralasin (inverse agonist mode, where Arg^2^ interacts with D281 and prevents CRS formation, see Figure 4). Other possibilities exist, including motion of the W84 indole rings (which are proximal to Phe^8^ in the crystal structure) to interfere with the Phe^8^ ring interaction with the salt bridge which is needed to reposition Arg^2^ away from D281 and promote CRS formation. The actions of inverse agonists, like sarilesin [and sartans, see below], derive partly from an inability to promote CRS formation, although the binding mode for these compounds enables coupling to arrestin and induces vasodilation, and may also signal receptor internalization, as the underlying mechanism for receptor desensitization.

### 2.5. Sartans: Nonpeptide Mimetics of AngII

Nonpeptide mimetics of AngII, referred to as ARBs (e.g., sartans), are inverse agonists, like saralasin and sarilesin. They have an important therapeutic role in cardiovascular medicine and are commonly prescribed for treating hypertension [80]. Binding of ARB sartans to AT_1_R has been studied using X-ray crystallography [81], and these compounds have been shown to interact within the same receptor binding pocket as peptide analogs, but with different contact residues. This overlapping binding site utilizes arginine R167 (rather than lysine K199 for AngII peptides) as the mainstay for the binding of nonpeptide ARBs [81,82] and apparently locks out AngII peptides from binding access to nearby K199. This illustrates that variations in binding site/mode (K199 or R167) can still promote coupling to arrestin and thereby produce inverse agonism. However, this leniency for interactive tendencies of ARBs may not apply to G protein coupling (agonism), since nonpeptide agonist versions of sartans have yet to be uncovered. Agonists act by inducing cooperativity of receptor dimers with G protein, leading to an increase in agonist affinity and the amplification of the receptor response [45], which is a more complex and discriminating mechanism than that producing inverse agonism and, therefore, much more difficult to mimic.

### 2.6. Bisartans: Second-Generation Nonpeptide Mimetics of AngII

Second-generation ARB bisartans [83,84], in which both imidazole nitrogens are substituted with biphenyltetrazole, have markedly increased binding affinity for AT_1_R, which may derive from the dual interaction of both tetrazoles with R167 [83] or perhaps simultaneously with R167 and K199. Pioneer research earlier on the design and synthesis of losartan analogs has led to the discovery of a new class of ARBs where the imidazole substituents, i.e., the butyl and hydroxy methylene groups at positions 2 and 4, respectively, are at reversed positions compared to losartan [85]. These analogs were the basis to further develop bisalkylated derivatives which symmetrically bear two biphenyl tetrazole groups on the two imidazole nitrogens, called bisartans, with notable properties relevant to hypertension and Coronavirus 2019 therapies [86,87].

The use of benzimidazole as a scaffold instead of imidazole and bis biphenyl tetrazole alkylation resulted in the development of new bisartans, which exhibited the unique binding affinities due to tetrazole and increased aromaticity reported in recent articles [88,89,90]. Interaction of aromatic phenyl groups with arginines, as between ARBs and AT_1_R R167, has been previously reported as a dominant binding factor due to the pi–pi electron interactions [91]. Arginine is a key amino acid in disease, and arginine blockers, like ARBs or bisartans containing warhead anionic tetrazoles, are emerging as promising pharmaceutics to battle arginine-based viruses and other diseases [84,89]. The unique and fascinating properties of tetrazole have recently received significant attention in medicinal chemistry for innovative therapies [92,93,94].

## 3. Materials and Methods

### 3.1. Steered Molecular Dynamics

The energetics of the proposed “Tyr → His → Phe” CRS pathway was explored by carrying out non-equilibrium SMD simulations of paired-residue deprotonation reactions. Two paired residue reactions were examined, including (i) proton transfer from the Tyr hydroxyl group (TyrOH^0e^) to the deprotonated His imidazole nitrogen group (TyrOH^0e^ + HisNN^−1e^ → TyrO^−1e^ + HisNNH^0e^) and (ii) proton migration from the fully-protonated His imidazole nitrogen (HisNNH^0e^) to the deprotonated Phe carboxylate group (HisNNH^0e^ + PheCOO^−1e^ → HisNN^−1e^ + PheCOOH^0e^). The reacting species (and associated QM calculations) were built in Hyperchem software version 8.0.10 (available online: http://hypercubeusa.com; accessed on 16 March 2025) and were positioned (*in vacuo*) with their respective reactive hydrogen bond pairs (e.g., TyrO[H]^0e^---NNHis^−1e^) separated by about 2–3 Å followed by system optimization using the unrestricted Hartree–Fock (UHF) RM1 semiempirical QM method. The Polak–Ribiere conjugate gradient method with a convergence of 0.1 kcal/mol-Å was used for all energy minimizations. Due to bond polarities inherent in the paired (donor–acceptor) reactive groups, a single hydrogen bond was formed between the residue species. Following optimizations, an SMD simulation was performed (in vacuo without periodic boundaries) by applying a directional acceleration bias (10.0 Å/ps^2^) to the acceptor residue. The SMD simulations were implemented using the RM1 semiempirical method (UHF calculation) known to emulate bond breakage and formation reactions in simple chemical systems [95,96,97]. The SMD run parameters included a time step of 1.0 fs, a constant temperature of 300 ^o^K, and a total run time of 122.5 fs, at which time the residues had become separated by 10–12 Å. The direction of the acceleration bias was aligned with the hydrogen bond longitudinal axis which resulted in the acceptor residue being pulled away from the donor residue. Quantities monitored over the SMD trajectories (recorded at 1.0 fs intervals) included the distance (in Å) between the donated hydrogen atom and the acceptor atom, total system potential energy (kcal/mol), and the total charge on each residue (from summation of partial atomic charges). Hydrogen bond rupture and formation were adjudicated by bond lengths. For example, if the donor proton closely tracked the acceptor atom of the leaving residue over the 122.5 fs SMD trajectory, the donor residue was considered to have been deprotonated with concomitant covalent bond formation between the donated proton and the acceptor atom. In both SMD reaction models (i.e., TyrO[H]^0e^---NNHis^−1e^ and HisNN[H]^0e^---OOCPhe^-1e^), the donor residue was deprotonated, with the donor proton forming a nascent covalent bond with the acceptor residue.

### 3.2. Calculation of Hydrogen Dissociation Energies

Relative static proton dissociation energies for TyrO_H_^0e^, HisNN_H_^0e^, and PheCOO_H_^0e^ were estimated based on a UHF semiempirical RM1 calculation of the residues in vacuo. Hydrogen dissociation energies were computed from the following relationship: AB_NRG_ = A_NRG_ + B_NRG_ + HBND_NRG_, where AB_NRG_ designates the energy (_NRG_) of the protonated parent residue (i.e., total system energy), A_NRG_ is the energy of the deprotonated residue at infinite separation from the hydrogen atom, B_NRG_ is the energy of the hydrogen atom at infinite separation, and HBND is the dissociation energy of the hydrogen bond (i.e., the interaction energy).

### 3.3. Ex Vivo Animal Experiments

#### 3.3.1. Materials

Human AngII sequence DRVYIHPF was purchased from Mimotopes (Mulgrave, VIC, Australia); CaCl_2_ (Cat#C1016), glucose (Cat#G7021), KCl (Cat#P9541), KH_2_PO_4_ (Cat#P0662), MgSO_4_·7H_2_O (Cat#230391), NaHCO_3_ (Cat#S5761), NaCl (Cat#S9888), phenylephrine hydrochloride (Cat#P1250000), and U46619 (Cat#538944) were purchased from Sigma Aldrich (St. Louis, MO, USA).

#### 3.3.2. Animals and Ethical Approval

Male C57BL/6 mice (*n* = 5) at 9 weeks of age and normotensive Wistar Kyoto rats at 4 weeks of age (*n* = 3) were purchased from the Animal Resource Centre (Perth, WA, Australia). All animals were housed at the Victoria University Werribee Campus Animal Facilities. Mice were housed 5 per cage until 12 weeks of age and rats were housed in pairs until 16 weeks of age. Animals were kept on a 12 h day/night circadian rhythm cycle and maintained at a constant temperature (21 °C) and relative humidity level (40 and 70%). Animals were fed a normal chow diet (Cat#SF00-100, Specialty Feeds, Glenn Forest, WA, Australia), and food and water were supplied ad libitum. All experimental procedures were conducted in accordance with the National Health and Medical Research Council “Australia Code of Practice for the Care and Use of Animals for Scientific Purposes” (8th edition, 2013; (8th edition, 2013, https://www.nhmrc.gov.au/about-us/publications/australian-code-care-and-use-animals-scientific-purposes) (accessed on 31 October 2024)), and were approved by the Victoria University Animal Ethics Committee (VUAEC#23/002 and 22/020).

#### 3.3.3. Anesthesia, Humane Dispatchment, and Artery Collection

Mice were first anaesthetized in a plastic induction chamber with gaseous isoflurane (flow rate: 4% in 1.5 L/min O_2_) for 5 min or until the righting reflex was absent. Mice were then placed into a mask for continued anesthesia exposure (flow rate: 2% in 0.8 L/min O_2_) for 5 min or until the absence of nail bed pain and corneal reflexes. Mice were then humanely dispatched via cardiac puncture by introducing a needle (23 G) into the left ventricle and collecting blood from the heart [61].

In studies using rats, there is evidence that isoflurane may desensitize [98] and inhibit [99,100] AngII-mediated contraction in arterial vascular smooth muscle cells by reducing calcium mobilization. Therefore, to maintain normal vascular responses to AngII, rats in our study were humanely dispatched by decapitation using a guillotine (Cat#DCAP-M, World Precision Instruments, Sarasota, FL, USA).

Due to the inability of AngII to invoke substantial contraction responses in mouse abdominal and thoracic aortae [58,59,60,61,79], iliac arteries were used in this study. After humane dispatch, a T-tube was introduced distal to the aortic arch to allow flushing of the iliac arteries with cold (4 °C) carbongenated (95% O_2_/5% CO_2_) Krebs solution (118 mM, NaCl; 4.7 mM KCl; 1.2 mM MgSO_4_·7H_2_O; 1.2 mM KH_2_PO_4_; 25 mM NaHCO_3_; 11.7 mM glucose; and 1.25 mM CaCl_2_) (pH: 7.4) [64]. The iliac arteries were retrieved from each animal and cleaned of connective and adipose tissue under a light microscope before being dissected into 2 mm rings in preparation for isometric tension myography [64].

#### 3.3.4. Isometric Tension Myography Studies

Iliac artery rings were immediately and sequentially placed into adjacent organ baths (OB8 and OB16, Zultek Engineering, Melbourne, VIC, Australia) containing 5 mL of Krebs–Henseleit solution, maintained at 37 °C and continuously bubbled with carbogen, and acclimatized for 30 min [64]. Rings were then gently mounted between two metal organ hooks attached to force displacement transducers to ensure that the endothelium was not accidentally damaged [64]. Rings were then slowly stretched to a physiologically relevant tension (rat: 0.3 g and mouse: 0.1 g [61]) and equilibrated for a further 30 min. Rings were then refreshed, re-stretched to original baseline tension, and acclimatized for a further 30 min. To investigate Ang II-induced contraction responses in iliac arteries, an AngII dose–response test (10^−8.0^–10^−5.0^ M, half Log units) was performed, with each cumulative dose being added after 2 min. Following the completion of AngII dose–response test, the rings were refreshed, allowed to return to baseline tension, and either contracted with high potassium physiological solution (rats) (125 mM/L KCl; 1.2 mM/L MgSO_4_·7H_2_O; 1.2 mM/L KH_2_PO_4_; 25 mM/L NaHCO_3_; 11.7 mM/L glucose; and 1.25 mM CaCl_2_) (pH: 7.4) [64] or U46619 (mouse) [2 × 10^–5.0^ M] (thromboxane analog) [61] to determine maximal standard contraction responses.

#### 3.3.5. Statistical Analysis

GraphPad prism (version 10.4.1, GraphPad Software Incorporated, La Jolla, CA, USA) was utilized to analyze data obtained from isometric tension analysis studies, and a two-way analysis of variance followed by Sidak’s multiple comparisons post hoc test was performed to determine significance. The significant *p* value was set at *p* < 0.05, and all data are represented as mean ± standard error of the mean (SEM).

## 4. Conclusions

The conformation of AngII in aprotic or low dielectric environments is characterized by the formation of an intramolecular CRS, which could be reenacted as an intermolecular CRS at the receptor in which the role of the Phe^8^ carboxylate of AngII is exchanged for the D281 sidechain at the receptor, and, in turn, the Phe^8^ carboxylate interacts with K199 of the receptor and determines agonist activity. For the AT_1_R-bound conformation of AngII, the availability of two options for the interaction of the Tyr^4^ hydroxyl of AngII with either the C-terminal carboxylate of AngII (bound in a salt bridge to K199 of the receptor) or the His^6^ imidazole of AngII (activated by D281 of the receptor) provides the opportunity for a gating mechanism for receptor signaling and biased agonism. Evidence suggests that CRS formation (Tyr^4^OH- -His^6^- -D281) may lead to agonist activation (vasoconstriction), whereas Tyr4 OH interaction with the Phe^8^ carboxylate in the salt bridge may result in inverse agonism (vasodilation, accompanied by prolonged receptor desensitization probably via receptor internalization). These findings have provided information enabling the design of the next generation of ARBs, such as bisartans.

## Figures and Tables

**Table 1:** Summary of the vasoactive response of various arteries from different species. Abbreviations: AngII, angiotensin II; AT_1_R, angiotensin II type 1 receptor; DRC, dose–response curve; h, hour; wks, weeks.

Species	Artery Type	AngII DRC	AngII_max_	Shape of Contraction	Reference
Patients undergoing coronary artery bypass graft surgery	Internal mammary artery	10^−10.0^–10^−6.0^ M	10^−6.5^ M	Parabola	[55]
Patients undergoing coronary artery bypass graft surgery	Internal mammary artery	10^−10.0^–10^−6.0^ M	10^−7.0^ M	Parabola	[56]
Healthy volunteers and patients undergoing elective cardiac revascularization surgery	Resistance arteries isolated from subcutaneous and gluteal adipose tissue	10^−10.0^–10^−7.5^ M	10^−8.0^ M and 10^−7.5^ M	Mix; however, reduced contraction responses to AngII when repeated on the same vessel 1 h after initial DRC, possibly through tachyphylaxis	[73]
Patients undergoing coronary artery bypass graft surgery with high and low cholesterol and C-reactive protein levels	Internal thoracic artery	10^−10.0^–10^−6.0^ M	10^−7.0^ M	Parabola	[57]
Patients undergoing coronary artery bypass graft surgery	Radial artery	10^−10.0^–10^−6.0^ M	10^−6.0^ M	Sigmoidal	[74]
Healthy and atherogenic male New Zealand White rabbits	Abdominal aorta	10^−9.0^–10^−6.0^ M	10^−7.5^ M	Parabola	[63]
Healthy male New Zealand White rabbits	Iliac artery	10^−11.0^–10^−5.0^ M	10^−8.0^ M	Parabola	[64,65,66]
Male Sprague–Dawley rats 1, 3, and 9 wks post-coronary artery ligation-induced myocardial infarction	Aorta	10^−10.0^–10^−6.0^ M	Sham: 10^−7.5^ M Myocardial infarction: 10^−7.5^ M and 10^−7.0^ M	Parabola	[67]
Rat	Juxta-medullary glomerular afferent and efferent arterioles	10^−12.5^–10^−5.5^ M	10^−7.0^ M	Sigmoidal	[75]
Normoglycemic and streptozotocin-induced Type I diabetic male Wistar rats	Carotid artery	10^−9.0^–10^−3.0^ M	10^−8.0^ M	Parabola	[69]
Male normotensive Wistar and spontaneous hypertensive rats	Abdominal aorta and iliac artery	10^−10.0^–10^−6.0^ M	10^−6.0^ M	Sigmoidal	[76]
Normoglycemic and streptozotocin-induced Type I diabetic male Wistar rats	Carotid artery	10^−11.0^–10^−6.0^ M	10^−7.0^ M	Parabola	[68]
Control and prenatal hypoxia exposure Sprague–Dawley rats	Middle cerebral artery	10^−11.0^–10^−5.0^ M	10^−5.0^ M	Sigmoidal	[77]
Male C57BL/6J mice	Abdominal aorta	10^−10.0^–10^−6.0^ M	10^−6.5^ M	Parabola	[58]
Female AT_1_Ra^+/+^ and AT_1_Ra^−/−^ mice	Abdominal aorta and femoral artery	10^−10.0^–10^−6.0^ M	10^−6.5^ M	Parabola	[59]
Male FVB/N mice	Mesenteric artery	10^−10.0^–10^−7.5^ M	10^−8.0^ M	Parabola	[60]
Old and young male C57BL/6J mice	Mesenteric artery	10^−11.0^–10^−8.0^ M	10^−8.0^ M	Sigmoidal	[78]
Fibulin-4^+/+^, Fibulin-4^+/-^, and Fibullin-4^-/-^ mice	Descending thoracic aorta, abdominal aorta, and iliac artery	10^−10.0^–10^−6.0^ M	10^−6.0^ M	Sigmoidal; however, it was reported that descending and ascending thoracic aortae did not respond to AngII at any concentration	[79]
Control and cisplatin-induced acute kidney injury male C57/BL6 mice	Brachiocephalic artery, iliac artery, and abdominal and thoracic aorta	10^−8.0^–10^−5.0^ M	10^−6.5^ M	Parabola; however, it was reported that the brachiocephalic artery and abdominal and thoracic aorta did not respond to AngII at any concentration	[61,62]

## Data Availability

The data underlying this study are not publicly available due to commercial and IP value. The data are available from the corresponding author upon reasonable request.

## Abbreviations

The following abbreviations are used in this manuscript:

ACE	Angiotensin-converting enzyme
AngII	Angiotensin II
ARB	Angiotensin receptor blocker
Arg	Arginine
Asp	Aspartic acid
AT_1_R	Angiotensin II type I receptor
Cat#	Catalog number
Cl^−^	Chloride
CRS	Charge relay system
H	Hydrogen
H-bond	Hydrogen bond
His	Histidine
HisNNH or HNNHis	His imidazole nitrogen (neutral, protonated)
Hr	Hour
Ile	Isoleucine
IL	Iliac artery
K^+^	Potassium
Leu	Leucine
Lys	Lysine
N	Nitrogen
Na^+^	Sodium
NOX	Nicotinamide adenine dinucleotide phosphate oxidase
O	Oxygen
OH	Hydroxyl
Phe	Phenylalanine
PheCCOO^−1e^	Phe with deprotonated carboxylate group
Pro	Proline
R	Sidechain
RAS	Renin–angiotensin system
ROS	Reactive oxygen species
Sar	Sarcosine
SEM	Standard error of mean
Ser	Serine
SMD	Steered molecular dynamics
Tyr	Tyrosine
TyrO^−1e^	Tyr with deprotonated phenol OH group
UHF	Unrestricted Hartree–Fock
Val	Valine
VUAEC	Victoria University Animal Ethics Committee
Wk	Week
Å	Angstrom
β-arrestin	Beta-arrestin
_NRG_	Energy
